# Marijuana Use and Stress Cardiomyopathy in the Young

**DOI:** 10.7759/cureus.18575

**Published:** 2021-10-07

**Authors:** Vivek Modi, Amitoj Singh, Jamshid Shirani

**Affiliations:** 1 Cardiology, St. Luke's University Health Network, Bethlehem, USA

**Keywords:** takotsubo cardiomyopathy, broken-heart syndrome, cardiac arrest, substance recreational use, stress induced cardiomyopathy, cannabis (marijuana)

## Abstract

Background

Increased accessibility, recreational use, and regional legalization of marijuana (cannabis) have been paralleled by widespread recognition of its serious cardiovascular complications (acute myocardial infarction, stroke, sudden death) particularly in the young. We aimed to examine trends in hospital admissions and outcomes of adults with stress cardiomyopathy (SC) in temporal relation to marijuana use.

Methods and results

A search of the 2003-2011 Nationwide Inpatient Sample (NIIS) database identified 33,343 admissions for SC of which 210 (0.06%) were temporally related to marijuana use. Demographics, clinical characteristics, and outcomes of marijuana users (MU) and non-marijuana users (NMU) with SC were compared. MU were younger (44±14 vs. 66±13 years), more often male (36% vs. 8%), and had lower prevalence of hypertension (38% vs. 62%), diabetes (2.4% vs. 17.6%), and hyperlipidemia (16% vs. 52%) while more often suffered from depression (33% vs. 15%), psychosis (12% vs. 4%), anxiety disorder (28% vs. 16%), alcohol use disorder (13% vs. 3%), tobacco use (73% vs. 29%), and polysubstance abuse (11% vs. 0.3%) [all p<0.001]. In addition, MU more often suffered a cardiac arrest and required placement of a defibrillator while congestive heart failure was more frequent in NMU. Logistic regression analysis on the entire database (n=71,753,900), adjusted for known risk factors for SC, identified marijuana use as an independent predictor of SC (OR=1.83; 95% CI=1.57-2.12, p<0.0001). Among MU, older age (>48 years) was a strong predictor of any major adverse cardiac event (OR=7.8; 95% CI=2.88-21.13; p<0.0001).

Conclusions

Marijuana use is linked to SC in younger individuals and is associated with significant morbidity despite being younger in age and having a more favorable cardiac risk factor profile in affected individuals.

## Introduction

Social acceptability, availability, and regional legalization of recreational marijuana (cannabis) have caused a dramatic increase in its use across the United States [1]. In 2014, 22.2 million individuals aged 12 years and older were considered current users of marijuana, and 4.2 million met the diagnostic criteria for abuse of or dependence on cannabis in the United States [2]. According to the 2014 National Survey on Drug Use and Health, marijuana remains the most commonly used illicit drug [3]. The sharp rise in recreational marijuana use has paralleled increased reporting of serious cardiovascular adverse events related to this substance [4-5]. These complications have ranged from acute myocardial infarction to ischemic stroke as well as cardiac arrhythmias and individual cases of stress cardiomyopathy (SC) [6-13]. We aimed to examine the temporal trends in incidence and outcomes of SC associated with marijuana use in a large national database of hospital admissions. In addition, we aimed to compare the findings to those patients who were admitted with a diagnosis of SC unrelated to the use of marijuana or other illicit drugs.

## Materials and methods

Data source

Data were obtained from the Nationwide Inpatient Sample (NIS) database, a part of the Healthcare Cost and Utilization Project sponsored by the Agency for Healthcare Research and Quality, for calendar years 2003 through 2011. The database contains discharge-level data for ~8 million hospital stays from ~1000 hospitals each year. It is designed to approximate a 20% stratified sample of community hospitals. A total of 46 states, representing ~96% of the United States population, participate in NIS. Hospital ownership, patient volume, teaching status, urban or rural location, and geographic region are used for stratified sampling; and discharge weights provided by the sponsor are used to obtain national estimates. The database is publicly available and contains de-identified information; therefore, the study was deemed exempt from institutional research board review.

Study population

All hospitalizations with a principal diagnosis (first or second diagnosis) of SC (transient ventricular regional ballooning or takotsubo) were included in the study. This was done using an International Classification of Diseases, Ninth Revision, Clinical Modification (ICD-9-CM) code 429.83. The study sample included a total of 33,343 patients. Among patients with SC, subsets of MU (n=210) and NMU (n=33133) were identified. Patients with marijuana use/abuse were identified using ICD 9-CM codes 305.2x and 304.3x. The two groups were mutually exclusive. Patient and hospital characteristics along with outcome parameters were compared between the two groups.

Patient and hospital characteristics

Baseline demographic and clinical features that were studied included both patient-level and hospital-level characteristics. Patient-level characteristics included demographics, primary payer, income quartile, all comorbidity measures for use with administrative data, other cardiovascular comorbidities (tobacco smoking, obesity, dyslipidemia, diabetes, known heart failure), drug abuse or dependence-related information other than marijuana (alcohol, cocaine, amphetamines, and hallucinogens), known risk factors for SC (pheochromocytoma, sepsis, hyperthyroidism, migraine, intracranial hemorrhage, seizure, depression, psychosis, anxiety disorder, and acute physical or emotional stress) and day of admission. Hospital-level characteristics included hospital location (urban or rural), hospital bed size (small, medium, or large), hospital region (Northeast, Midwest, South, or West), and teaching versus non-teaching status. A list of ICD-9-CM software codes used to identify patients, demographics, clinical characteristics, co-morbidities, in-hospital procedures, complications, and outcomes are provided in the Appendix.

Outcome measures

The outcome measures evaluated were in-hospital mortality, cardiogenic shock, cardiac arrest, acute systolic or diastolic congestive heart failure, intra-aortic balloon pump use, cardioverter-defibrillator implantation, discharge to a facility other than home, length of stay, cost of hospitalization, and major adverse cardiovascular events (MACE). The latter was defined as in-hospital mortality, length of hospital stay exceeding four days, acute heart failure, cardiogenic shock, and discharge to a facility other than home.

Statistical analysis

Weighted data were used for all statistical analyses. Results were expressed as numbers (%) for categorical variables and mean ± standard deviation for continuous variables. Differences between groups were analyzed with the use of the student’s t-test for continuous variables and the χ2 test for categorical variables, respectively. Logistic regression was used to compare in-hospital outcomes among study groups. The regression model was adjusted for demographics, hospital characteristics, all comorbidity measures for use with administrative data, as well as other clinically relevant co-morbidities and complications that were deemed as important. Adjusted odds ratio (OR) and 95% confidence intervals (CI) were used to report the results of logistic regression. A two-tailed p-value less than 0.05 was considered statistically significant. Statistical analyses were performed using SPSS statistical software version 20.0 (IBM Corp., Armonk, New York). Comparison of the length of hospital stay between the two groups was performed using the independent samples Mann Whitney U test.

## Results

Patient characteristics

The study population consisted of 33,343 patients admitted from 2003 through 2011 for SC, of whom 210 (0.06%) were related to active marijuana use. As shown in Table 1, marijuana users (MU) were significantly younger than non-marijuana users (NMU) (44±14-vs-66±13 years) and were more often male (36% vs. 8%) and non-white (29% vs. 16%) [all p<0.001]. In addition, the prevalence of all cardiac risk factors except tobacco use was significantly lower in MU compared to NMU. MU, however, suffered more frequently from psychosocial conditions and more often abused alcohol and illicit drugs. Overall, illicit drugs other than marijuana were used by 11.4% of MU compared to 0.3% of NMU (p<0.001). Post-traumatic stress disorder was reported in a minority of NMU (0.4%) and none in the MU. While several neurologic disorders, in particular, migraine headaches, ischemic stroke, and seizures were more often reported in MU, they tended to have a lower prevalence of most systemic conditions other than liver disease, chronic pulmonary disease, and coagulopathies.

**Table 1:** Comparison of demographic, psychosocial, and clinical characteristics of marijuana and non-marijuana users admitted to hospital with stress cardiomyopathy * Items used in multivariable regression; NS: not significant

Variable		Marijuana use		P-value
		Yes (n=210)	No (n=33133)	
Demographic information				
Age (years)		44±14	66±13	<0.001
Female		64%	92%	<0.001
Race	White	71%	84%	<0.001
	Black	23%	6%	<0.001
	Hispanic	3%	5%	NS
	Asian	0%	1.5%	NS
	Other	3%	2.5%	NS
Cardiovascular risk factors and diseases				
Hypertension*		38%	63%	<0.001
Diabetes mellitus*		2.4%	17.6%	<0.001
Hyperlipidemia*		15.7%	47.6%	<0.001
Smoking*		73%	29%	<0.001
Morbid obesity*		0	1.9%	0.036
Valvular heart disease		0	1.9%	0.038
Chronic congestive heart failure	Systolic	0	2.5%	0.02
	Diastolic	0	0.3%	NS
Psychosocial factors				
Depression*		33%	15%	<0.001
Psychosis		12%	4%	<0.001
Anxiety disorder*		28%	16%	<0.001
Acute stress*		2.4%	1%	0.06
Alcohol abuse or dependence*		13%	2.8%	<0.001
Cocaine abuse or dependence*		9.5%	0.2%	<0.001
Amphetamine abuse or dependence*		2.4%	0.1%	<0.001
Hallucinogen abuse or dependence		0	0	-
Neurologic conditions				
Migraine headache*		13%	2%	<0.001
Transient ischemic attack/stroke*		7.6%	2.8%	<0.001
Intracranial bleed		0	0.1%	NS
Subarachnoid bleed*		0	0.1%	NS
Subdural bleed		0	0.05%	NS
Seizure*		5.2%	1%	<0.001
Other neurologic diseases		8.1%	6.5%	NS
Paralysis		2.4%	0.9%	0.049
Systemic and other organ system conditions				
Sepsis*		0	1.1%	NS
Hypothyroidism		2.4%	16.3%	<0.001
Hyperthyroidism*		0%	0.6%	NS
Liver disease		7.2%	1.3%	<0.001
Renal failure		1.9%	5.3%	0.028
Fluid and electrolytes abnormalities		21%	19%	NS
Acute or chronic venous thromboembolism*		0	0.7%	NS
Peripheral vascular disease		0	6.3%	<0.001
Human immunodeficiency virus infection		0	0.1%	NS
Collagen vascular disease		9%	4.3%	0.003
Blood loss anemia		0	0.7%	NS
Chronic pulmonary disease		38%	22%	<0.001
Coagulopathy		4.7%	2.1%	0.016
Pulmonary circulation disease		0	0.9%	NS
Solid tumor without metastasis		2.4%	1.5%	NS
Metastatic disease		0	1%	NS
Peptic ulcer disease excluding gastrointestinal bleed		0	0.02%	NS
Pheochromocytoma		0	0	NS

Admission characteristics

**Figure 1:**
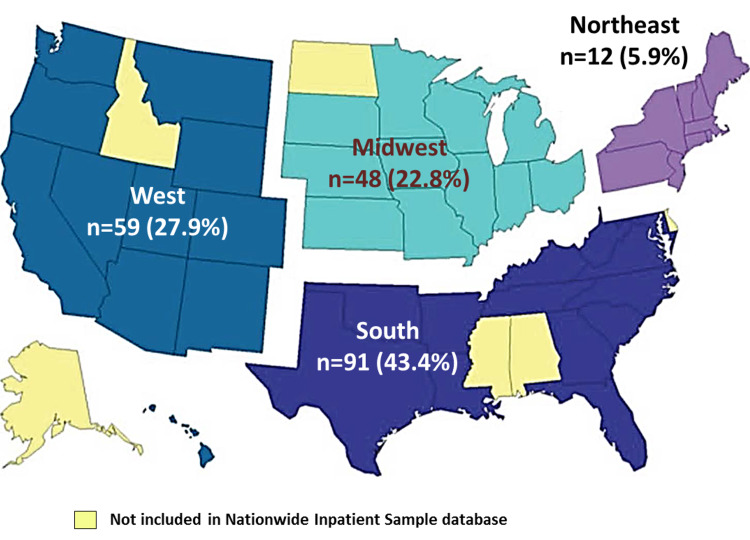
Map of the United States showing the distribution of hospital admissions for stress cardiomyopathy in temporal relation to marijuana use between 2003 and 2011 based on represented regions in the Nationwide Inpatient Sample (NIS) data

Table 2 compares the median adjusted income, insurance status, hospital and admission characteristics, as well as length of stay and cost of hospitalization among MU and NMU. As shown, MU had a substantially lower income and was more often either uninsured or insured through Medicaid. Most MU (71.3%) were admitted to southern and western states (Figure 1) and were more likely to present in an emergent situation to urban teaching hospitals. While the length of hospital stay was similar in the two groups, the median cost of hospitalization was ~$7207 higher per patient in the MU group. There was a statistically significant increase in the annual rate of SC admissions related to marijuana use during the study period (OR=1.520; 95% CI=1.403-1.647; p<0.0001).

**Table 2:** Comparison of income, insurance, hospital, and cost characteristics among marijuana and non-marijuana users admitted to hospital with stress cardiomyopathy NS: not significant

Variable		Marijuana use		P-value
		Yes (n=210)	No (n=33133)	
Income and Insurance characteristics				
Primary payer	Medicare	15.7%	56.5%	<0.001
	Medicaid	14.9%	5%	<0.001
	Private insurance	32%	32.5%	NS
	Self-pay	22.8%	3.4%	<0.001
	No change	3.5%	0,3%	<0.001
	Other	11.9%	2.3%	<0.001
Income quartile	1^st^	38.5%	22.4%	<0.001
	2^nd^	25.9%	24.3%	NS
	3^rd^	15.7%	26.8%	<0.001
	4^th^	19.9%	26.5%	<0.001
Hospital and admission characteristics				
Region of hospital	Northeast	5.9%	19.9%	<0.001
	Midwest	22.8%	26%	NS
	South	43.4%	33.2%	0.002
	West	27.9%	20.9%	0.012
Admission type	Emergency	74.1%	63.6%	0.02
	Urgent	20.4%	28%	NS
	Elective	5.4%	8.2%	NS
	Trauma	0%	0.2%	NS
Weekend admission		23.7%	23.3%	NS
Bed size	Small	11.9%	8%	NS
	Medium	16.9%	22.1%	NS
	Large	71.2%	69.9%	NS
Teaching status	Non-teaching	34.2%	37.8%	0.01
	Teaching	63.4%	54.2%	0.01
Hospital cost ($)		34319±30807	27112±36663	<0.001
Length of stay (days)		3.53±3.2	3.71±4	NS

Outcomes

**Figure 2:**
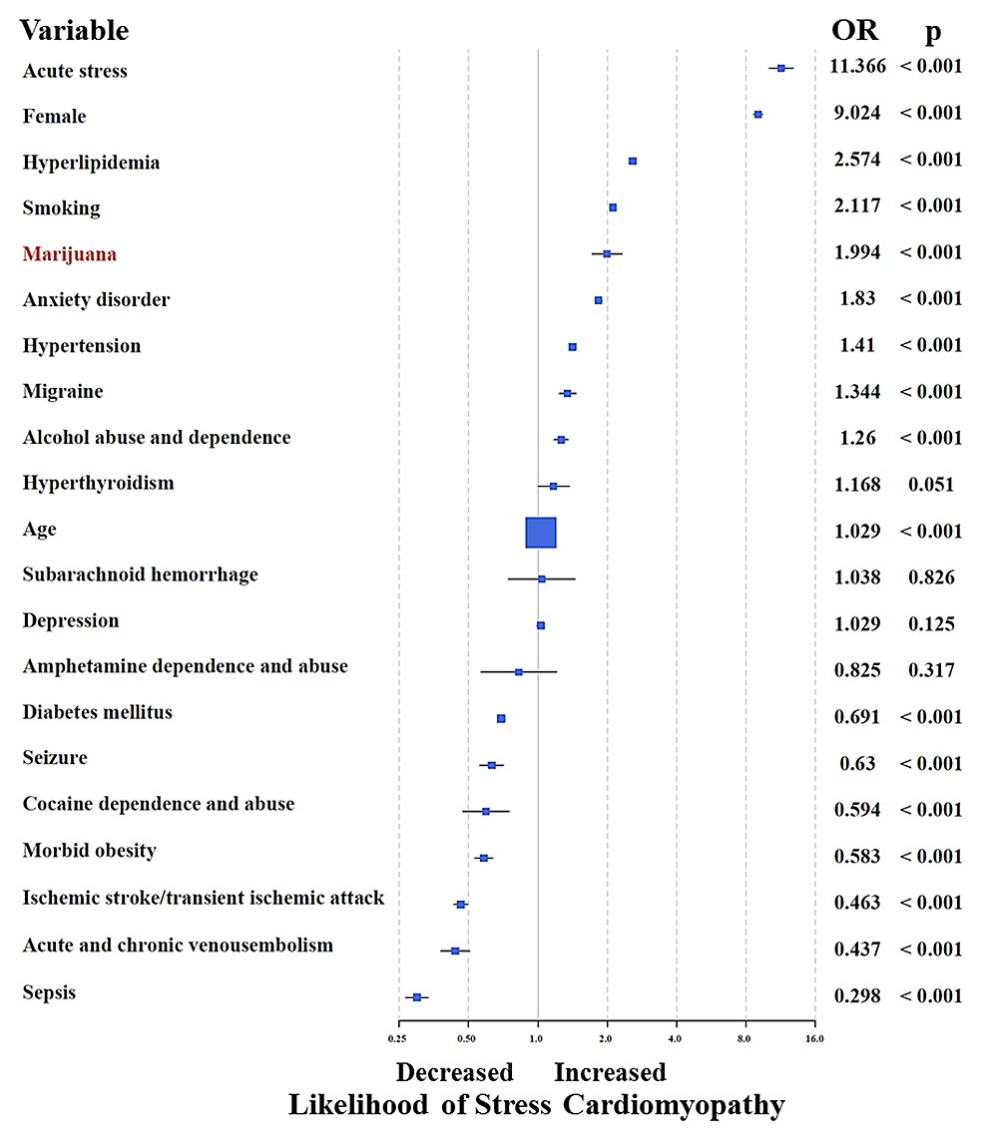
Multivariable binary logistic regression analysis on the entire database (n=71,753,900), adjusted for known risk factors for stress cardiomyopathy, showing marijuana use as an independent predictor of stress cardiomyopathy

**Figure 3:**
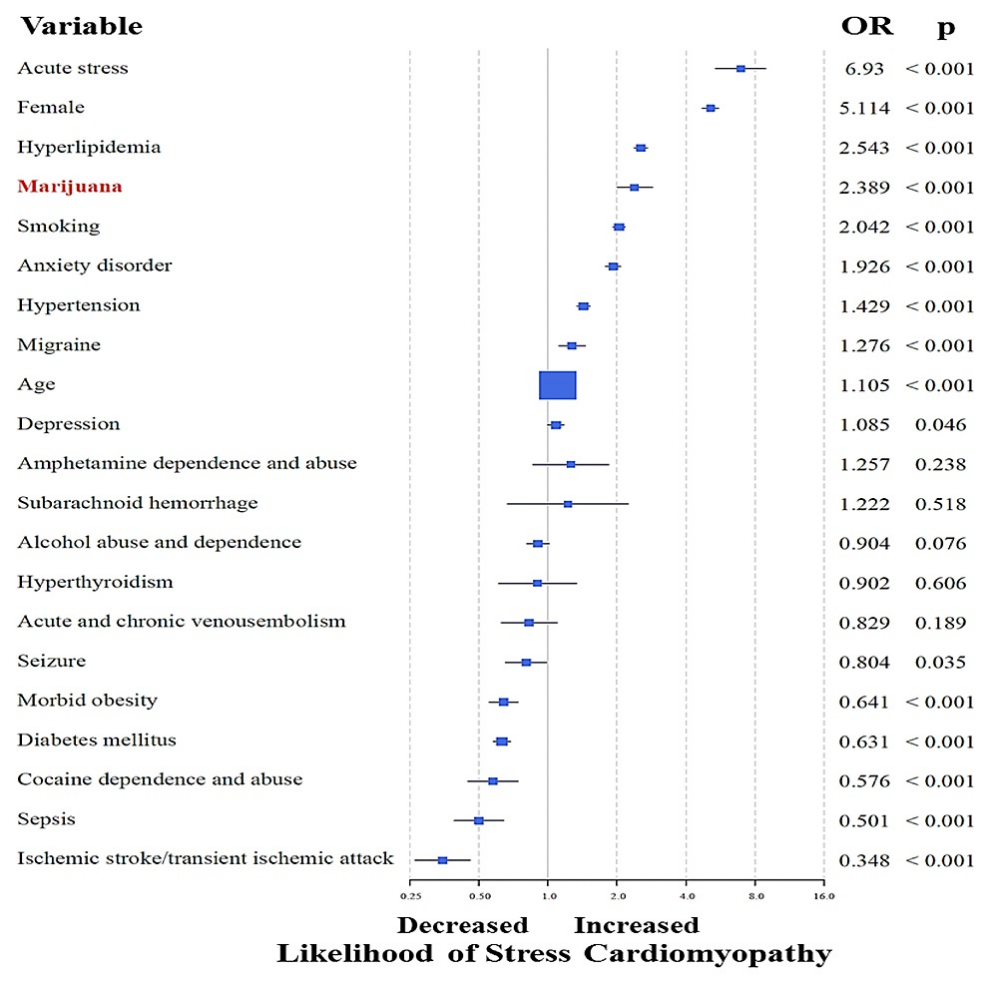
Multivariable binary logistic regression analysis on the younger individuals (15-54-year-old) in the entire database, adjusted for known risk factors for stress cardiomyopathy, showing marijuana use as an independent predictor of stress cardiomyopathy

Although no mortality was observed, a total of 51 other serious adverse events were recorded in MU (0.243 events per subject) including acute stroke in 11 (5.2%), acute heart failure in eight (4.0%), cardiogenic shock in six (3.0%), and cardiac arrest in five (2.4%) (Table 3). In addition, an intra-aortic balloon pump and automatic implantable cardioverter-defibrillator (AICD) were needed in six (3.0%) and five (2.4%) MU, respectively. When compared to NMU, the frequency of acute stroke, cardiac arrest, and AICD insertion were significantly higher among MU. Despite a significantly younger age, MU suffered a similar frequency of serious adverse events compared to NMU. However, NMU were more often discharged to a facility other than home. Multivariable binary logistic regression analysis on the entire database (n=71,753,900), adjusted for known risk factors for SC, identified marijuana use as an independent predictor of SC (OR=1.994; 95% CI=1.716-2.317; p<0.001) (Figure 2). The strength of this association was more appreciable among younger (15-54 year old) subjects (OR=2.389; 95% CI=2.004-2.848; p<0.001) (Figure 3).

**Table 3:** Comparison of cardiovascular outcomes of marijuana and non-marijuana users admitted to hospital with stress cardiomyopathy * Includes all listed serious adverse events; NS=not significant

Outcomes	Marijuana use		P-value
	Yes (n=210)	No (n=33133)	
Mortality	0	1.02%	NS
Cardiogenic shock	3%	2.5%	NS
Cardiac arrest	2.4%	0.8%	0.034
Acute systolic congestive heart failure	4%	6.7%	NS
Acute diastolic congestive heart failure	0	0.6%	NS
Acute systolic and diastolic CHF	4%	7.3%	NS
IABP use	3%	1.9%	NS
ICD implantation	2.4%	0.6%	0.008
Discharge to a facility other than home	4.8%	10%	0.011
Acute Stroke	5.2%	0.9%	<0.001
Number of adverse events per subject*	0.243	0.251	NS

Comparison of younger and older patients

The median age for MU was 48 years. Comparison of younger (≤48 years old, n=106) and older (n=104) MU revealed significant differences in baseline characteristics (Table 4). In particular, older MU were exclusively female, more often Caucasian, and had a higher prevalence of hypertension and smoking while less likely to have diabetes or obesity. The older cohort, however, more often suffered from several chronic psychological and systemic illnesses, including depression, migraine headaches, ischemic cerebrovascular events, collagen vascular disease, and chronic obstructive pulmonary disease. A comparison of in-hospital cardiovascular outcomes of younger and older MU is shown in Table 5. While younger MU more often had a cardiac arrest or received an AICD, older patients had significantly higher rates of cardiogenic shock, acute heart failure, acute stroke, or need for IABP support.

**Table 4:** Comparison of demographic, psychosocial, and clinical characteristics of younger (≤ 48) and older (>48) marijuana users admitted to hospital with stress cardiomyopathy * Items used in multivariable regression NS=not significant

Variable		Age (years)		P-values
		≤48 (n=106)	>48 (n=104)	
Demographic information				
Age (years, median)		32.9±9.8	54±4.6	-
Female		29.1%	100%	<0.001
Race	White	68.2%	73.8%	0.005
	Black	19.8%	26.2%	NS
	Hispanic	6%	0%	0.03
	Other	6%	0%	0.03
Cardiovascular risk factors and diseases				
Hypertension*		23.4%	52.9%	<0.001
Diabetes mellitus*		4.8%	0	0.03
Hyperlipidemia*		13.9%	17.8%	NS
Smoking*		60.4%	86.8%	<0.001
Obesity*		14.4%	4.3%	0.009
Psychosocial factors				
Depression*		14.8%	51.3%	<0.001
Psychosis		9.8%	14.1%	NS
Anxiety disorder*		24.6%	32.2%	NS
Acute stress*		4.8%	0	0.03
Alcohol abuse or dependence*		18.2%	8.8%	0.048
Cocaine abuse or dependence*		9.3%	9.4%	NS
Amphetamine abuse or dependence*		4.8%	0	0.03
Neurological Conditions				
Migraine headache*		0	26.6%	<0.001
Transient ischemic attack/stroke*		0	14.9%	<0.001
Seizure*		4.8%	6.1%	NS
Other neurologic diseases		10.1%	6.1%	NS
Paralysis		4.8%	0	0.03
Systemic and other organ system conditions				
Hypothyroidism		0	4.8%	0.02
Liver disease		5.3%	9%	NS
Renal failure		0	4.3%	0.04
Fluid and electrolytes abnormalities		18.9%	23.3%	NS
Collagen vascular disease		0	18.4%	<0.001
Chronic pulmonary disease		20.9%	54.5%	<0.001
Coagulopathy		4.8%	4.8%	NS
Solid tumor without metastasis		0	4.8%	0.02

**Table 5:** Comparison of cardiovascular outcomes of younger (≤48) and older (>48) marijuana users admitted to hospital with stress cardiomyopathy NS: not significant; IABP: intra-aortic balloon pump; ICD: implantable cardioverter-defibrillator

Outcomes	Age of MU (years)		P-value
	≤48 (n=106)	>48 (n=104)	
Mortality	0	0	-
Cardiogenic shock	0	6.1% (6)	0.012
Cardiac arrest	4.8% (5)	0	0.03
Acute systolic congestive heart failure	0	8.1% (8)	0.004
Acute diastolic congestive heart failure	0	0	-
Acute systolic and diastolic CHF	0	8.1% (8)	0.004
IABP use	0	6.1% (6)	0.012
ICD implantation	4.8% (5)	0	0.03
Discharge to a facility other than home	0	9.2% (10)	0.001
Acute stroke	0	10.4% (11)	0.0006
Number of adverse events per subject	0.09	0.47	<0.001

## Discussion

The findings of the current study add to an increasing body of information that has linked the recreational use of marijuana to serious adverse cardiovascular events in the young [4-14]. These serious events have included acute myocardial infarction, acute ischemic stroke, cardiac arrest, and sudden unexpected death likely secondary to ventricular dysrhythmias. Marijuana-related SC has been uncommonly reported previously [10-14]. However, our data indicate that the condition is likely significantly underreported partly due to the wide geographic scatter of encountered cases. It is also concerning that the number of admissions for SC among marijuana users has steadily increased over the recent years. More widespread use of marijuana and increases in the potency of the active components of cannabis may have contributed to that observation [15-16]. Despite young age and relatively low rates of cardiovascular risk factors, SC did not follow a benign in-hospital course in MU. In fact, cardiogenic shock, cardiac arrest, and acute heart failure occurred in 19 patients, and intra-aortic balloon pumps and implantable cardioverter-defibrillators were required in 11 of those patients. In addition, patients stayed up to 17 days in the hospital and 10 patients had to be transferred to long-term skilled nursing facilities after discharge. The relatively high incidence of complications and lengthy hospital stays may have accounted for a significantly higher cost of hospitalization in MU compared to NMU with SC (an average difference of >$7,000 per patient).

Cardiovascular effects of marijuana use

Marijuana is composed of dried and shredded leaves, seeds, and flowers of the plant Cannabis sativa. It contains a host of alkaloids of which delta-9-tetrahydrocannabinol (THC) and cannabidiol (CBD) are the most chemically active. These active ingredients interact with the cannabinoid CB1 and CB2 receptors [17]. The receptors are predominantly inhibitory G-protein-coupled membrane-bound receptors that activate intracellular signal transduction pathways. While CB1 receptors are present in major organs. including the heart, vasculature, and autonomic nervous system, CB2 receptors are predominantly located in the cells of the immune system. Stimulation of the CB1 receptor, the primary target of THC, is shown to promote oxidative stress, inflammation, endothelial injury, as well as activation of the sympathetic and inhibition of the parasympathetic nervous system [18]. The net effect of THC on the cardiovascular system has been reviewed recently and includes a hyperadrenergic state, heightened oxidative stress, pro-coagulation, and depressed myocardial contractility [18]. These physiologic changes explain the increased likelihood of acute coronary syndrome, lethal arrhythmias, stroke, arteriopathy, and SC following exposure to THC [18].

Marijuana and SC

An association between cannabis use and SC has been reported in few cases [10-14]. The present study indicates that the prevalence of such an association is likely underestimated at least in part due to the widely scattered occurrence of individual cases. The pathophysiology of SC in cannabis users has not been fully understood. However, evidence for a direct role of the cannabinoid system in the pathogenesis of SC has been accumulating. THC is shown to exert a myocardial suppressant effect through CB1 receptors [19]. In addition, cannabis use has been shown to cause a hyperadrenergic state through receptor-mediated and receptor-independent mechanisms [20-22]. The central role of catecholamines in the pathogenesis of SC has been proposed based on current observations [23-24]. It has been, thus, speculated that SC may represent a form of neurogenic myocardial stunning as a result of the centrally triggered release of catecholamines from sympathetic nerve terminals in the myocardium with subsequent cardioinhibitory and coronary vasospastic effects [25]. Based on similarities observed on brain imaging during stress and after exposure to THC, it has also been proposed that amygdala-centered neuronal circuits may underlie the pathogenesis of SC in MU [26]. Regardless of the underlying pathogenic mechanisms, our data indicate that SC in MU is not a benign condition and is associated with serious adverse outcomes in this group of mostly younger individuals.

Marijuana use and pre-existing heart disease

The presence of pre-existing myocardial dysfunction may predispose individuals to deterioration of left ventricular systolic function following the use of potent synthetic cannabinoids [27]. In patients with chronic stable angina, smoking a single marijuana cigarette decreases exercise time to angina by 48% and reduces left ventricular stroke volume and ejection fraction [28]. Decreased myocardial oxygen supply in such a setting occurs by a combination of increased vascular tone due to sympathetic stimulation and elevated carboxyhemoglobin after smoking marijuana [28]. In a population study (Determinants of Myocardial Infarction Onset Study), marijuana was identified as a trigger for acute coronary syndrome [29]. In the latter study, the risk of acute myocardial infarction increased nearly five times within an hour of using marijuana [29]. In individuals who already had a previous acute myocardial infarction, marijuana use more than once a week was associated with a threefold increase in mortality [29]. In the present study, older individuals with marijuana-induced SC had a substantially higher rate of cardiovascular risk factors.

Study limitations

Our study is retrospective in design and thus cannot establish causation. We cannot tell based on this database whether marijuana was inhaled or orally ingested by the users. Finally, this database does not allow the determination of the quantity of marijuana used.

## Conclusions

The incidence of marijuana-induced SC is on the rise. Marijuana use is linked to SC in a distinct cohort of younger individuals and is associated with significant morbidity despite younger age and a more favorable cardiac risk factor profile compared to SC in non-users of Marijuana. The data also raises significant questions regarding the safety of marijuana use in older individuals with pre-existing cardiovascular diseases.

## Appendices

**Table 6:** A list of International Classification of Diseases, Ninth Revision, Clinical Modification (ICD-9-CM) codes used to identify patients, demographics, clinical characteristics, co-morbidities, procedures, complications, and outcomes

Variable	ICD-9-CM Software Code(s)
Marijuana dependence or abuse	305.2x, 304.3x
Takotsubo	429.83
Hyperlipidemia	272x
Smoking	305.1, V158.2
Hyperthyroidism	242.x
Morbid obesity	278.01
Acute and chronic venous thromboembolism	453x
Alcohol abuse and dependence	305x, 303x, 291x
Cocaine abuse and dependence	304.2, 304.20, 304.21, 304.22, 305.6, 305.60, 305.61, 305.62
Amphetamine abuse and dependence	305.7, 305.70-72 304.4, 304.40-42
Hallucinogen abuse and dependence	305.3, 305.30, 305.31-32, 304.50-52
Anxiety disorder	309.24, 309.28, 300x
Acute Stress	308x
Depression	300.4, 301.12, 309.0, 309.1, 311
Pheochromocytoma and hyper-functioning adrenal states	255.6, 194.0, 227.0
Sepsis	995.92, 785.52, 790.7, 117.9, 112x, 115x, 036.2, 038x
Migraine	346x
Ischemic Stroke or transient ischemic attack	433x-436x
Subdural and extradural hemorrhage	432x, 439
Intracerebral hemorrhage	431
Subarachnoid hemorrhage	430
Seizure	780.3, 780.39
Implantable cardioverter-defibrillator (ICD) insertion	37.94
Biventricular Pacemaker with ICD	00.51
Biventricular Pacemaker	00.50
Pacemaker implantation	37.70-37.74
Acute systolic congestive heart failure	428.41, 428.43
Acute diastolic congestive heart failure	428.31, 428.33
Chronic systolic congestive heart failure	428.22
Chronic diastolic congestive heart failure	428.32
Cardiac arrest	427.5
Cardiogenic shock	785.51
Intra-aortic balloon pump (IABP) insertion	37.61
Mechanical circulatory support other than IABP	37.52, 37.60, 37.62, 37.65, 37.66, 37.68
Cardiac transplantation	37.51
